# Wearable Multisensor Ring-Shaped Probe for Assessing Stress and Blood Oxygenation: Design and Preliminary Measurements

**DOI:** 10.3390/bios13040460

**Published:** 2023-04-05

**Authors:** Simone Valenti, Gabriele Volpes, Antonino Parisi, Daniele Peri, Jinseok Lee, Luca Faes, Alessandro Busacca, Riccardo Pernice

**Affiliations:** 1 Department of Engineering, University of Palermo, Viale delle Scienze, Building 9, 90128 Palermo, Italy; 2 Department of Biomedical Engineering, Kyung Hee University, Yongin 17104, Republic of Korea

**Keywords:** wearable health devices (WHD), photoplethysmography (PPG), galvanic skin response (GSR), oxygen saturation (SpO_2_), biomedical devices

## Abstract

The increasing interest in innovative solutions for health and physiological monitoring has recently fostered the development of smaller biomedical devices. These devices are capable of recording an increasingly large number of biosignals simultaneously, while maximizing the user’s comfort. In this study, we have designed and realized a novel wearable multisensor ring-shaped probe that enables synchronous, real-time acquisition of photoplethysmographic (PPG) and galvanic skin response (GSR) signals. The device integrates both the PPG and GSR sensors onto a single probe that can be easily placed on the finger, thereby minimizing the device footprint and overall size. The system enables the extraction of various physiological indices, including heart rate (HR) and its variability, oxygen saturation (SpO_2_), and GSR levels, as well as their dynamic changes over time, to facilitate the detection of different physiological states, e.g., rest and stress. After a preliminary SpO_2_ calibration procedure, measurements have been carried out in laboratory on healthy subjects to demonstrate the feasibility of using our system to detect rapid changes in HR, skin conductance, and SpO_2_ across various physiological conditions (i.e., rest, sudden stress-like situation and breath holding). The early findings encourage the use of the device in daily-life conditions for real-time monitoring of different physiological states.

## 1. Introduction

In recent years, wearable health devices (WHDs) have gained increasing popularity thanks to their ability to accurately monitor an ever-growing number of parameters. The scientific community is nowadays pushing towards realizing wearable WHDs that integrate smaller, more accurate, and less power-consuming sensors. Many worldwide companies in the tech sector have, in fact, produced wristband-based devices that provide information in real time about heart rate, oxygen saturation, and the quality of sleep (e.g., Apple Watch, Galaxy Watch, Xiaomi Mi-band, etc.) [1,2,3]. The latest technological advancements in the microelectronics sector have the potential to allow the production of devices even smaller than watches that can be suitably located on different body districts. For example, rings [4] can include small sensors based on the photoplethysmographic (PPG) technique to extract physiological information on the cardiovascular system [5,6,7,8,9,10].

This non-invasive, comfortable and easy-to-use approach is preferred in place of the classic and less comfortable techniques, such as electrocardiography (ECG), as it allows researchers to measure cardiovascular parameters in a more practical way (e.g., heart rate (HR), HR variability (HRV) [11,12,13]) that can provide useful information about the individual’s physiological state. In particular, it is widely known that short-term rhythms in HRV measurements are generated through the interactions between autonomic neural activity, blood pressure, and respiratory control systems, providing fundamental information about autonomic tone that reflects the appropriate dynamic change in nervous activity [14,15,16]. Therefore, small and lightweight PPG probes can be integrated within portable biosensing devices to measure cardiovascular parameters, providing useful information to monitor an individual’s heart health status. Furthermore, the concurrent utilization of dual-wavelength PPG sensors and appropriate signal processing algorithms enables the extraction of oxygen saturation level of hemoglobin in the arterial blood, providing valuable information for monitoring individuals affected by respiratory-related pathologies, such as sleep apnea or COVID-19 [17,18,19,20,21]. These conditions can lead to low oxygenation levels that pose a potentially life-threatening risk. While oxygen saturation (SaO_2_) values can be extracted invasively by means of arterial blood gas test, the method based on PPG relies on the distinct response of oxygenated and deoxygenated hemoglobin to different wavelengths and typically yields values only slightly different from SaO_2_ [10,22,23].

In addition to the blood pulsation activity, another important signal that can be acquired from the finger where the PPG sensor is placed is the so-called galvanic skin response (GSR); this signal measures conductance and reflects autonomic changes in the electrical properties of the epidermal tissue due to the activity of the sweat glands, each one innervated by several sudomotor fibers [24,25]. This signal reflects the fact that the human organism reacts to a stressful event (either mental or physical) by activating the sympathetic nervous system (SNS), which produces sweating in several body districts, including the fingers. This defense mechanism is also known as general adaptation syndrome (GAS) and produces sudden changes in different physiological parameters (e.g., increased HR, blood pressure and sweating). Such changes are due to both sympathetic and parasympathetic (PNS) branches of the autonomic nervous system (ANS), intervening to prepare the individual to react and overcome the stressor [26]. While many literature works have demonstrated that stress can be properly estimated using only HRV [12,27,28], recent studies evidenced that GSR can be considered an excellent real-time correlation of stress, being linearly related to arousal [29,30,31]. Some researchers regard GSR as the foremost real-time indicator of stress, even superseding HRV measures [29,30], or suggest combining GSR as a measure of SNS activity and the high frequency HRV spectral component as a measure of PNS activity [32]. Therefore, stress detection accuracy can be enhanced through a multimodal approach that utilizes machine learning techniques to classify stress conditions based on a combination of both HRV and GSR indices, instead of relying solely on HRV measures [31,33,34].

In this study, we have exploited the aforementioned concepts to design and realize a new wearable device that allows the simultaneous acquisition of data from a PPG and a GSR sensor, both placed on the same body location (specifically, the finger). The prototype, which takes the form of a ring-shaped probe, demonstrates the practicality of conducting synchronous acquisition of PPG and GSR signals on the finger, as well as extracting SpO_2_ levels to enable the concurrent evaluation of an individual’s physiological stress state and oxygen saturation. This approach obviates the need to utilize additional devices positioned on other parts of the body, instead consolidating all sensors in one location, thus reducing the device footprint and increasing the individual’s comfort during measurements. The device is a noteworthy advancement if compared to the architecture of our previous prototype of portable electronic system, which was not wearable and more bulky [35]. To the best of our knowledge, this is the first-ever ring-shaped device capable of synchronously acquiring PPG and GSR signals, allowing it not only to assess cardiovascular status and characterize the SNS activity (through HRV and GSR analyses), but also to detect low oxygenation levels potentially dangerous for the individual’s health (especially useful during the pandemic emergency period). To verify the proper functioning of the ring-shaped probe in monitoring the variations in the acquired signals, a multiparametric acquisition was finally conducted on a cohort of healthy subjects during rest, a stress-inducing task, and breath-holding exercises.

## 2. Materials and Methods

### 2.1. Description of the Realized Wearable Biosensing System

**Figure 1:**
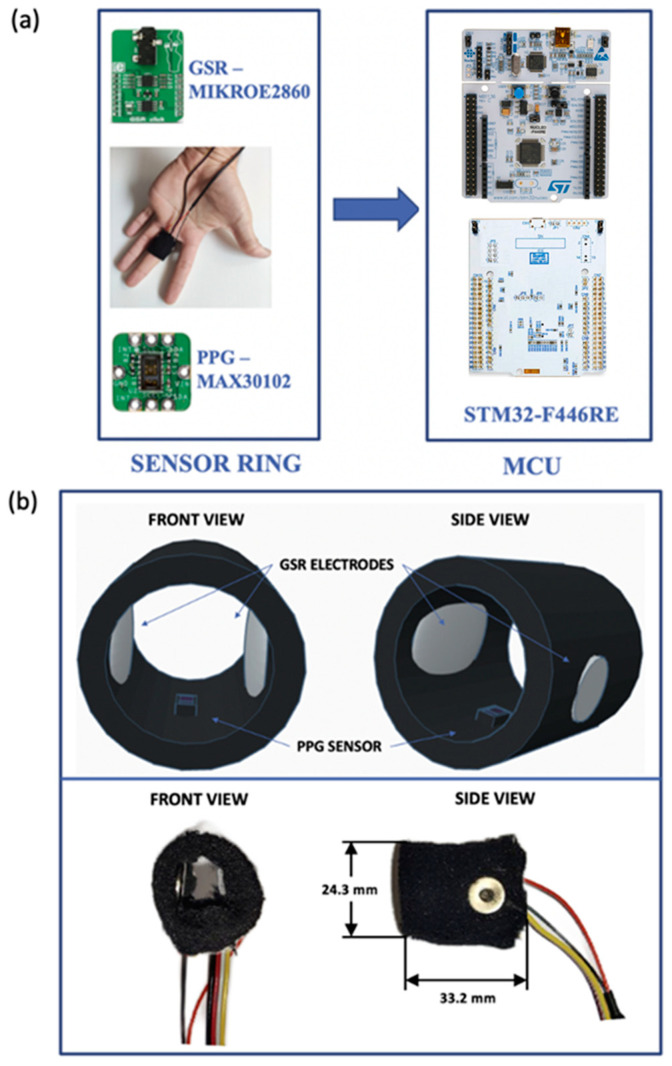
(**a**) Block diagram of the acquisition system. Both PPG and GSR electrodes were placed inside the elastic band (sensor ring) and transferred the data to the microcontroller unit (MCU). (**b**) Front and side views of the design (**top**) and implementation (**bottom**) of the proposed ring-shaped probe, including its dimensions.

The system realized in this study (shown in Figure 1a) was composed of two parts: (1) a compact and lightweight ring-shaped sensor probe, which contained both PPG and GSR sensors and consisted of an non-toxic and allergen-free elastic band appositely designed to be comfortably worn on the finger; (2) a microcontroller-based system, which managed the ring sensor and received, preprocessed and transferred the acquired data to a personal computer (PC). The elastic band and the location of the sensors were appositely chosen to be worn well tight to the finger to maximize the pressure contact with the skin (and standardize it among subjects), but without creating discomfort for the user.

Figure 1b shows the front and side views of both the design sketch (top panel) and of the implementation (bottom panel) of the ring probe. To make the probe suitable to be worn on a finger, a cylindrical body comprising elastic fabric was exploited to wrap both the GSR electrodes and the PPG sensor. The external dimensions of the probe were 33.2 × 24.3 mm, as presented also in Figure 1b. Due to design requirements, the two electrodes, each one having a diameter of 14.7 mm, were placed on two diametrically opposite points of the cylindrical body to allow the integration of the PPG sensor, reducing space waste. The PPG sensors, having a size of 3.3 × 5.6 mm, were positioned in the center of the probe to acquire the PPG signal at the bottom side of the finger, this being the most suitable location on the finger to achieve the best quality of the recorded waveform.

As regards the acquisition of the GSR signal, we used two Ag/AgCl (silver-chloride) electrodes placed on the ring probe and connected to the Mikroe-2860 GSR-Click sensor (manufactured by MikroElektronika), which exploited an analog front-end circuit based on the volt-amperometric method for the detection of the skin resistance measurements. In particular, a DC voltage was applied to the first electrode, while the second one was connected to a R = 100 kΩ resistor which, together with the resistance offered by the epidermal tissue, formed a voltage divider circuit. By measuring the voltage across the resistor, it was possible to derive the current flowing through both resistances and, as a result, determine the skin resistance. For this reason, once the raw signal was filtered through a passive low-pass filter with a cut-off frequency of f = 15.9 Hz, the residual potential of the second electrode was sampled by a low-noise 12-bit analog-to-digital converter (ADC) and, finally, sent to the microcontroller via the serial peripheral interface (SPI) communication protocol [36]. Finally, the skin resistance was obtained by computing the voltage difference between the electrodes, divided by the current flowing on the resistor.

A MAX30102 chip (produced by Maxim Integrated) was employed for the acquisition of the PPG waveform in reflection mode. This choice was carried out to put the photodetector and the LEDs on the same side, thus leaving more room on the opposite side for further sensors in future upgrades of the device. Moreover, reflectance PPG allowed us to noticeably reduce the dependence of the acquired signal on the tissue volume [37]. The LEDs integrated within MAX30102 were dual-wavelength, i.e., emitted light at both 660 nm (red) with 6.5 mW of radiant power and 880 nm (infrared) with 9.8 mW of radiant power. The MAX30102 also integrated an analog front-end for the ambient light cancellation and a high-resolution (up to 18-bit) ultra-low noise ADC, with typical dark current in the built-in photodiode of 0.01% of full-scale and power consumption of about 1 mW (which can be further reduced using the ultra-low power modality) [38]. The immunity of the front-end to the ambient light was checked through preliminary measurements carried out, firstly, in dark ambient conditions and then by simulating the sunlight with an Oriel Sol3A solar simulator (manufactured by Newport), with the output set at 0.8 sun, i.e., a typical value in a sunny day condition. The measurement results showed the excellent behavior of the ring probe, detecting only a slight percentage increase in the DC components of the PPG red (0.17%) and infrared (0.5%) waveforms. No changes were reported for the AC components, thus validating our probe for acquiring PPG signals even in the presence of a strong ambient light.

Finally, the STM32F446RE Nucleo-64 development board (manufactured by STMicroelectronics, Geneva, Switzerland) was used for the management of the sensors. GSR data were acquired by connecting the analog output of the Mikroe-2860 sensor directly to the STM32-F446RE 12-bit ADC and operating in continuous sampling mode. The MAX30102 sensor was configured to acquire both the 660 and 880 nm PPG waveforms sampled with the internal ADC every 1.25 ms, exchanging data with the microcontroller through the inter-integrated circuit (I^2^C) communication protocol. To ensure the synchronous acquisition of both signals, the STM32F446RE was programmed to acquire data whenever MAX30102 ended the analog-to-digital conversion. The proper synchronization was guaranteed by the MAX30102 external interrupt occurring every sampling period when the sensors data were already available on the register.

The system was designed to be low-power, with the estimated total power consumption for all the embedded devices and conditioning circuitry being around 205 mW. Although our system supports sampling frequencies up to 1.6 kHz, for the preliminary measurements herein carried out a sampling frequency of f = 800 Hz was set, with 17-bit ADC resolution; this frequency is considered appropriate for heart rate variability analyses [39].

### 2.2. Experimental Protocol

Measurements were carried out on the middle finger of the left hand of five normotensive, healthy volunteers (3 males; age 25 ± 2.3 years) monitored in a sitting position while undergoing an experimental protocol specifically aimed at evoking sympathetic activation and, thus, producing changes in both heart rate and sweat glands activity, as occurring during stress-like situations. During the protocol, the subjects were kept still, with the hands on the table to reduce motion artifacts. The experimental protocol performed by the subjects consisted of the following five phases:

- Rest 1 (R1): the subject under test watched relaxing videos (nature, sea, sunset). The duration of this phase was 180 s;
- Sudden fright (SF): the subject experienced a sudden vision of a jump scare video (with the aim of arousing fright). The duration of this phase was 10 s;
- Rest 2 (R2): the subject watched another relaxing video to re-establish a resting condition and allow the complete recovery of the physiological parameters. The duration of this phase was 180 s;
- Breath holding (BH): the subject was asked to hold his breath for 40 s;
- Rest 3 (R3): the subject started to breathe again normally to allow the complete recovery of the physiological parameters. The duration of this phase was 180 s.

### 2.3. Data Processing

An appositely developed graphical user interface was implemented using MATLAB R2021b© (The MathWorks, Inc., Natick, MA, USA) to show and pre-process in real time the acquired signals, while also computing the average HR and SpO_2_ values. After a visual inspection for motion artifacts, the acquired data were stored for further off-line post-processing.

The PPG waveforms were first filtered using a zero-phase 4th order lowpass Butterworth digital filter (cut-off frequency: 8 Hz), while a 100-point moving average filter was employed to smooth the GSR signal. The PPG maxima peaks were extracted using a MATLAB threshold-based peak detection algorithm to obtain the pulse–pulse interval (PPI) time series. The PPG minima were also extracted to be used for HRV indices and SpO_2_ computation alongside with maxima. The instantaneous HR was calculated from the PPI minima time series as HR = 60/PPI [bpm]. Time–domain HRV analysis was performed computing the average (MEAN) and the standard deviation of the normal-to-normal beats (SDNN) of the PPI minima series values [12]. Since the indices were extracted from PPG signals, hereinafter we will refer to pulse rate variability (PRV), which has been widely recognized in the literature as a surrogate of HRV with good agreement in most physiological states [13,40]. MEAN and SDNN indices were computed on the whole durations of each phase of the experimental protocol (R1, SF, R2, BH, R3) to assess variations among different physiological conditions.

**Figure 2:**
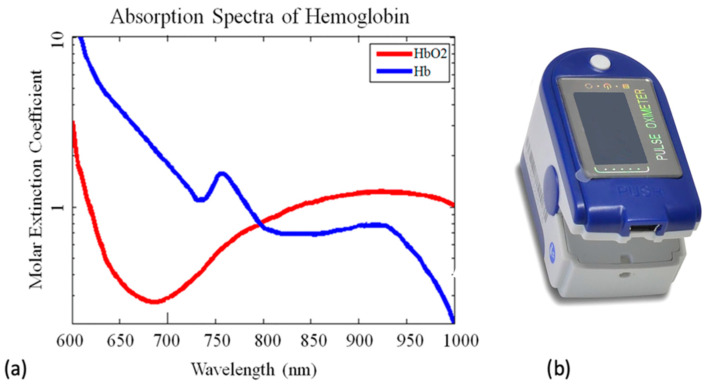
(**a**) Absorbance curve of oxygenated and deoxygenated hemoglobin (readapted from [43]). (**b**) Frontal view of SpO_2_ Pulse Oxygen in Blood Sensor PRO for MySignals (eHealth Medical Development Platform) manufactured by Libelium and used for SpO_2_ calibration [44].

The SpO_2_ values were computed exploiting the fact that the absorbance of a red blood cell coincides with that of a hemoglobin solution [41]. This “transparency” of erythrocytes allowed measurements of oxygen saturation, expressed as the percentage of hemoglobin that is saturated by oxygen (typically included between 95% and 100% at sea level) [42]. For this purpose, it is possible to take advantage of the knowledge of the variation in the absorption spectrum of hemoglobin in relation to its degree of saturation by oxygen, shown in Figure 2a [43].

Since the isosbestic point of the hemoglobin spectrum is located at 805 nm (see Figure 2a, crossing of the blue and red curves) a pulse oximeter usually exploits two light emitting diodes (LEDs) emitting both a red light (typically with wavelength λ = 650–670 nm) and an infrared light (with λ = 870–890 nm), used in combination with a receiving photodiode.

After acquiring the two optical pulse waves from both the red and the infrared LEDs, it was possible to express the so-called ratio of ratios “*R*” as [45]:

(1) $$R=\frac{\frac{AC_{RED}}{DC_{RED}}}{\frac{AC_{IR}}{DC_{IR}}}.\;$$

*R* is a ratio of ratios of the pulsatile and non-pulsatile components of red-to-IR light absorption; the light absorption of two wavelengths is thus derived from the pulsatile-added volume of oxygenated arterial blood [46,47].

The two components of the PPG signal (i.e., AC and DC) were evaluated starting from the extraction of the minimum and maximum peaks of the red (RED) and infrared (IR) waveforms. In particular, the DC component was evaluated as the mean value between the maximum and minimum peaks of the pulse wave signal, while the AC component was evaluated as the difference between the two peaks. A widely employed linear empirical equation for computing SpO_2_ is the following [48]:

(2) $$SpO_{2\;}=a+b*R\;,$$

where *a* and *b* are two constants to be determined through calibration according to the optical characteristics of the adopted device. In this work, calibration was carried out using a commercial pulse oximeter, the SpO_2_ Pulse Oxygen in Blood Sensor PRO for MySignals (eHealth Medical Development Platform, manufactured by Libelium [44]; see Figure 2b). This device allowed us to acquire heart rate from 25 to 250 bpm and SpO_2_ from 35% to 100%. The device had an accuracy of 2% and a resolution of 1% within the SpO_2_ range of interest (i.e., between 80 and 100%) [49].

**Figure 3:**
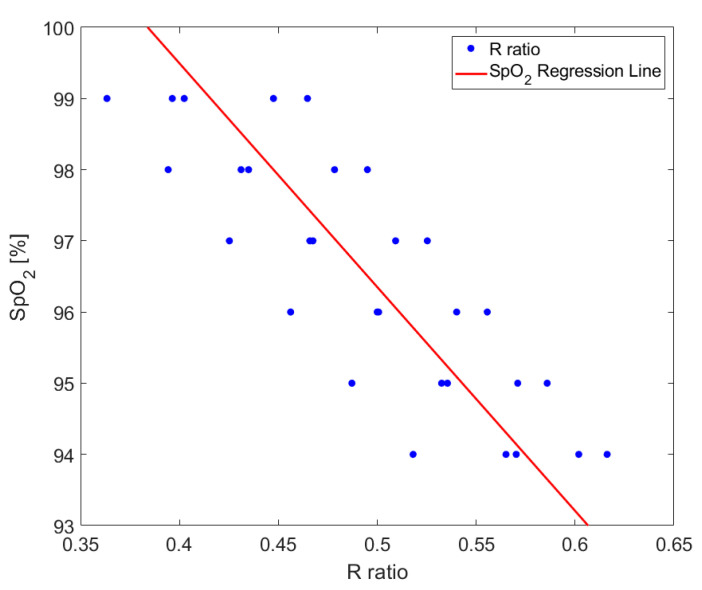
Scatter plot of the 25 data pairs of R to SpO_2_ used for calibration (blue dots) and the computed SpO_2_ regression line (red line).

The calibration procedure of our device was carried out on preliminary measurements from the volunteers to extract the *a* and *b* constants of Equation (2) from a calibration curve obtained by curve fitting. During the breath holding protocol, simultaneous measurements of the red and infrared PPG waveforms were carried out with the ring-shaped probe and the commercial pulse oximeter, respectively. The R values for SpO_2_ percentages ranging from 94 to 99% (i.e., maximum and minimum SpO_2_ values detected during the whole measurement protocol) were calculated and depicted in Figure 3. Finally, the regression line between the R and SpO_2_ values was computed using the least squares approximation method by curve fitting the data from volunteers [48,50]. The obtained regression line was:

(3) $$SpO_{2\;}=112.07-31.44R$$

After calibration, we compared the average values of the SpO_2_ samples acquired using our system to those recorded using the pulse oximeter. We discovered that our device accuracy falls within the range (2%) of the commercial device used as the reference. The goodness of fit was evaluated computing the widely employed root mean square error (RMSE) metric, obtaining RMSE = 1.11.

As previously mentioned, in this work we have also acquired the GSR signal, which varies during a stressful event due to the GAS, which is the activity of the sweat glands directly proportional to the SNS activation level. The GSR signal can be divided into two components: the skin conductance level (SCL) and the skin conductance response (SCR). SCL is the measure of the basal level of the sweat glands activity (i.e., the sweating without stressors), which depends on the individual’s physical characteristics, and is considered as a reference level to evaluate sweating variations. SCR (i.e., the phasic activity) is a highly variable signal that detects sudden changes of sweating following stressful events, reflecting a fast variation in the autonomic arousal. Acquiring both components is therefore essential to obtain a high-quality GSR signal for which a correct detection of the SNS activity is guaranteed. In our analysis, once the GSR signal was correctly acquired using the sensor and ADC front-end described in Section 2.1, both SCL and SCR components were extracted employing the MATLAB-based toolbox LEDALAB; this software allowed us to separate the two components, exploiting the continuous decomposition analysis based on a standard deconvolution method [51]. According to this approach, the extraction of tonic and phasic activity was carried out through deconvolution of GSR data. The phasic activity was assumed to superimpose a slowly varying tonic activity (SCL). Sudomotor nerve activity results in sweat secretion, thus triggering a specific variation in skin conductance. In mathematical terms, the sudomotor nerve activity is considered as a driver, which generates a sequence of mostly distinct impulses that are the sudomotor nerve bursts (i.e., the SCR peaks). The phasic driver component exhibited a virtual-zero baseline and distinct phasic responses. Further details and the mathematical formulation of this method are reported in Benedek and Kaernbach [51].

In this work, to assess variations among different physiological conditions, we evaluated the mean values of the GSR level and SCL component computed on the whole durations of each phase of the experimental protocol (R1, SF, R2, BH, R3) [52]. Finally, the number of peaks of the SCR component was also computed considering 10-s windows before and after each phase transition [52]. This choice considered the number of generated peaks in relation to the level of cognitive and emotional stress [53].

## 3. Results

Herein, we present the preliminary results demonstrating the effectiveness of our system for acquiring physiological signals of interest (i.e., PPG, GSR) as well as extracting cardiovascular markers that vary according to an individual’s stress level.

**Figure 4:**
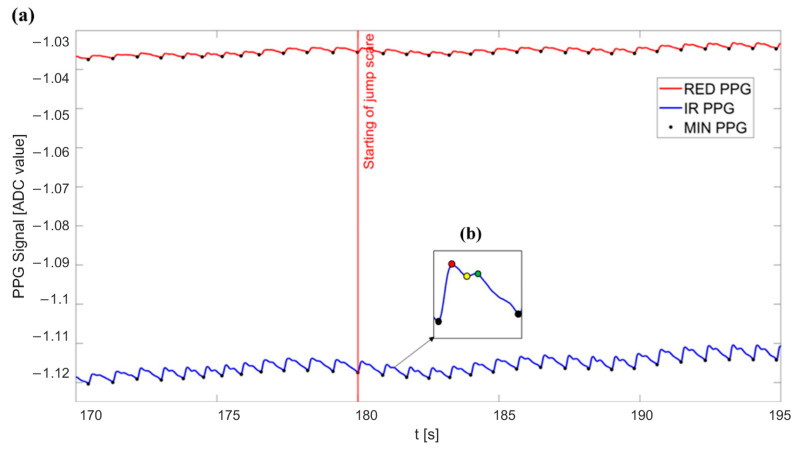
(**a**) 25-s excerpt of the PPG signals acquired on a subject during the experimental protocol. Signals are represented as raw ADC values. Please also note that for visualization purposes the signals were inverted during post-processing to reflect a typical PPG waveform. The red- and blue-colored curves indicate the PPG signals acquired using red and infrared light, respectively. Black dots represent the minimum value of each PPG waveform, detected for each cardiac cycle to measure the PPI intervals. The vertical red line denotes the transition between the R1 and SF phase. (**b**) The inset shows a zoom of an exemplary PPG waveform acquired with our device. In the inset: systolic foot (black dots), systolic peak (red dot), diastolic notch (yellow dot), and diastolic peak (green dot).

Figure 4 shows the two PPG waveforms (i.e., using red and infrared wavelengths) during a 25-s window ranging from second 170 to 195 of one acquisition on a subject, corresponding to the transition from the R1 phase to the SF phase. No movement artifacts were reported during the acquisitions as the experimental protocol was appositely implemented to avoid any movements of the subjects. In the inset, a zoom of an exemplary PPG waveform acquired with our device is shown. As reported, the waveform clearly exhibits all the main fiducial points of a PPG signal, i.e., the systolic foot (representing the beginning of the systolic heart phase), the systolic peak (representing the maximum blood volume during the systolic phase), the dicrotic notch (corresponding to the closure of the aortic valve prevalently seen in the descending phase of young subjects with healthy compliant arteries [54]), and the diastolic peak (corresponding to both the diastolic heart phase and the peripheral wave reflection). Given that all the fiducial points are visible, the acquired signal can be considered as having a “diagnostic quality” according to Moscato et al. [55], thus allowing a more in-depth morphological analysis if compared to “basic” quality signals.

**Figure 5:**
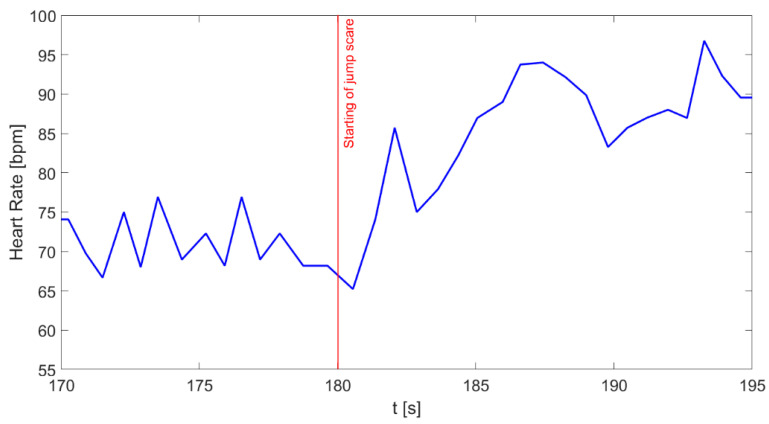
Beat-to-beat heart rate during the analyzed 25-s window extracted from PPG signal. The vertical red line marks the transition between the R1 and the SF phase.

The instantaneous HR (shown in Figure 5) was extracted from the minimum values of the red PPG signal during each period. At the beginning of the SF condition, a noticeable HR increase can be observed, which continues for the whole duration of the phase.

**Figure 6:**
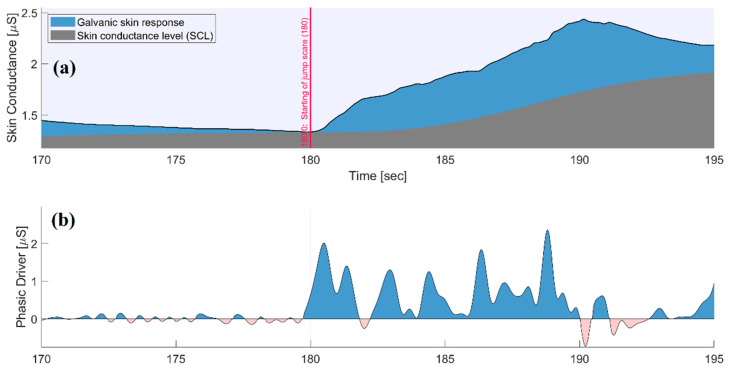
25-s window of the GSR signal acquired during the experimental protocol and extraction of its components through LEDALAB toolbox. (**a**) GSR signal (blue area and black line) and SCL component (grey area); (**b**) SCR component in terms of phasic driver. The vertical red line denotes the transition between the R1 phase and the SF phase.

Figure 6a depicts the GSR waveform in the same 25-s window. A sudden change in the GSR signal occurs at the start of the SF phase, indicating an increase in the skin conductance (i.e., decrease in skin resistance). The decomposition analysis carried out by LEDALAB (shown in Figure 6b) highlights both a general increase in tonic activity (SCL in Figure 6a), with especially intense phasic activity of the GSR signal that begins precisely in correspondence of the visualization of the jump scare video (Figure 6b). The increased number of SCR peaks (also larger in amplitude) in Figure 6b is indicative of the sudomotor nerve bursts generating sweat secretion, which can be attributed to the visualization of the frightening video.

**Figure 7:**
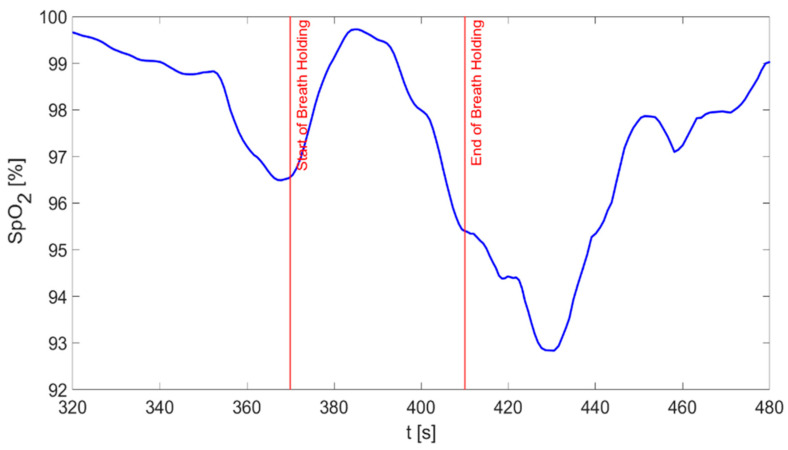
Instantaneous values of SpO_2_ during a 160-s window. The vertical red lines delimitate the 40-s BH phase.

Figure 7 shows the values of oxygen saturation during a 160-s window including the 40-s BH phase of the experimental protocol. A 3-point average (i.e., smoothing) digital filter was applied to the SpO_2_ waveform to better visualize the trend of this parameter over the recorded time. As reported, a few seconds after the subject starts holding their breath, oxygen saturation begins to decrease; such reduction persists a few seconds after the restart of normal breathing, evidencing a delay in the physiological response of the organism.

Table 1 reports the results of time domain PRV and GSR parameters (expressed as mean value ± standard deviation) computed on all the subjects involved in the measurement campaign, for each phase of the experimental protocol. To allow a direct comparison of the skin conductance indices (i.e., GSR and SCL) among subjects, the minimum value reached for each subject during the entire acquisition has been subtracted.

The results reported in Table 1 confirm a decrease in mean PPI (i.e., increased HR) and an increase in GSR and SCL mean values during SF. Opposite trends are observed during the transition from R2 to BH; this transition was characterized by an increased PPI mean, reflecting a slightly reduced heart rate due to respiratory sinus arrhythmia. Conversely, GSR and SCL mean values show a tendency to rise again, albeit to a lesser extent compared to the SF phase. The number of peaks of the SCR component were also computed, taking into account 10-s windows before and after each physiological condition transition, i.e., window W1: last 10 s in R1 phase, W2: first 10 s during SF phase; W3: first 10 s in R2 phase; W4: last 10 s in R2 phase; W5: first 10 s in BH phase. The results reported in Table 2 highlight an increased number of peaks (i.e., increase in the phasic activity) during SF and (to a smaller extent) during BH compared to the preceding resting conditions.

## 4. Discussion

The results of the experiment carried out with the novel ring-shaped probe confirm the proper working of the device and the feasibility of the proposed multiparametric acquisition for the characterization of different physiological responses. The lower values of the heart rate (Figure 5) and the GSR signal (Figure 6) during the R1 phase confirm that the subject was relaxed; lower values are also evidence of the dominance of the parasympathetic activity typical of resting conditions. After the vision of the scary video, an increase of 32% (from 72 bpm to 95 bpm) for the instantaneous values of the heart rate (Figure 5) and of 67% (from 1.45 µS to 2.42 µS) for the GSR (Figure 6) immediately after the phase transition suggests a shift in the sympathovagal balance towards dominance of the SNS; this trend is typical of stress conditions [56,57].

These trends are also confirmed by observing the time–domain PRV measures and the skin conductance indices averaged on all the subjects, computed on each of the five phases (Table 1 and Table 2); the decrease in mean PPI and the increase in the number of SCR peaks during SF are reported on all the subjects, thus evidencing a strong sympathetic activation. The same observation applies to the GSR and SCL indices during the transition from R1 to SF phase. The irregular trend of the heart rate reported in Figure 5 suggests a strong variability in the time distance between two successive PPG peaks, which becomes even more marked during the SF phase, as also evidenced by the SDNN index in Table 1.

On the other hand, opposite trends are reported when moving from R2 to BH: the increased PPI mean, i.e., a slightly reduced heart rate, is related to respiratory sinus arrhythmia [58,59]. The GSR and SCL mean values again tend to rise, even if not as much as during SF, thus suggesting that breath holding protocol may elicit increased sweating. Nonetheless, in contrast to the transition from R1 to SF phase, this trend is not observed in all subjects under consideration. These findings suggest that the breath-holding phase elicits heterogeneous responses among subjects and does not induce a strong sympathetic activation; this trend is also evidenced by the slightly lower heart rate recorded. This contrasts with the previous case and suggests a more complex interplay between physiological mechanisms underlying the response to BH phase.

The decreasing trend in SpO_2_ values during and up to a few seconds after the end of the BH phase (Figure 7) highlights that the experimental protocol is influencing the oxygen saturation of the subjects and, at the same time, confirms that the device and the SpO_2_ extraction algorithm correctly allow the monitoring of SpO_2_ values. From a physiological point of view, the delay observed at the phase transitions with regard to SpO_2_ trends is evidence that the variations in oxygen saturation are a slow phenomenon, as already reported in Scully et al. [60]. Conversely, the declining trend observed before the start of the protocol and the rising trend within the initial seconds of the BH phase suggest that the phenomena underlying the variations of SpO_2_ are not only very complex from a physiological point of view, but also subject-specific.

Overall, the reported results confirm the feasibility of employing our ring-shaped wearable device to synchronously acquire different biosignals. The use of a high-performance microcontroller together with lightweight and compact commercial sensors makes our device comfortable, cost-effective and easy to implement as a scalable but fully wearable solution. The compact design of the device (in terms of its shape, size, and weight) minimizes the space occupied on the body, thus significantly reducing any discomfort experienced by the user. Moreover, the measurements carried out highlighted the high-quality of the acquired signals, with particular regard to the PPG waveform that exhibits all the main fiducial points used in literature, thus paving the way for the exploitation of our system for further morphological analyses [55].

The aforementioned features make our system suitable for a wide range of scenarios, encompassing not only home monitoring of frail individuals and fitness applications but also clinical settings. Additionally, the combined utilization of our prototype alongside with other medical devices has the potential to prove beneficial for the early diagnosis of cardiovascular diseases. In this regard, the synchronous acquisition of ECG and pulse waves may enable the timely detection of pathological changes, including microcirculatory dysfunction at both coronary [61,62] and peripheral arteries [63,64]. The simultaneous measurement of HR and SpO_2_ indices is crucial for detecting cardiovascular parameters and blood oxygenation levels in physical activity scenarios—namely, pre-, during-, and post-exercise stages. Within this context, the concomitant measurement of post-exercise heart rate and oxygen consumption could enable the detection of exercise-induced arrhythmia due to heart rate and protein kinase level alterations [65].

Nonetheless, a major limitation of our system is that the preliminary version does not implement robust motion–artifact detection or removal algorithms. Even though in this study an experimental protocol that avoids the movements of subjects has been used, future activity should focus on the onboard implementation of algorithms to exploit the already embedded gyroscope/accelerometer modules for detecting or, even better, removing the motion artifacts occurring when using the device during daily-life activities [66]. Another limitation may reside in the peak detection and filtering procedures employed in this study; filtering can induce shifts of the location of PPG feature points and lead to inaccuracy in the computed PRV indices, especially when using PPG peaks [67,68]. For this reason, in future work feature points that are more robust against the filtering-induced time shift effects should be extracted (e.g., maximal first derivative, inflection points) to increase accuracy; this has previously been suggested in several studies [9,67]. Finally, another limitation is that the time–domain indicators for PPG waveforms used in this work have not been validated through the extraction of HRV indicators from synchronous measurements of ECG signals.

## 5. Conclusions

In this study, a prototype of a wearable biomedical device capable of performing synchronous acquisition of PPG and GSR signals has been realized and tested. Moreover, an algorithm for the instantaneous measurement of SpO_2_ values has been developed and calibrated. Preliminary results carried out in a laboratory environment indicate that the developed system can be suitably employed for monitoring physiological states and assessing both the emotional arousal and the oxygen saturation. These encouraging preliminary results pave the way for testing our system in larger measurement campaigns, specifically during daily-life activities. Such campaigns will also encompass the simultaneous acquisition of ECG signals through a previously realized portable multisensor device, aimed to further validate the PRV indices with HRV measures and assess cardiorespiratory interactions [35,69].

The implementation of more standardized measurement protocols capable of eliciting different stress types (e.g., orthostatic stress, mental stress, fatigue) and levels are envisaged as a future activity to better assess the reliability and the limitations of the system; these protocols will also allow for a more comprehensive characterization of ANS in various physiological states. To achieve this objective, well-established HRV/PRV measures in the time domain, as well as in the frequency and information-theoretic domains [12,13,70], should be leveraged and computed in real time directly within the firmware of our device. The real-time assessment of stress levels conducted through a wearable device can pave the way to improve anxiety management through the so-called HRV biofeedback [71]. Finally, a further upgrade of the prototype should also foresee the integration within a single compact board of both the sensors and the microcontroller, together with other wearable sensors (e.g., body temperature sensor) potentially useful for a more complete health status assessment or for motion artifact removal [66,72,73,74].

## Figures and Tables

**Table 1:** Results of time domain PRV and GSR parameters (expressed as mean value ± standard deviation).

Measure/Phase	R1	SF	R2	BH	R3
PPI mean [ms] PPI SDNN [ms]	815 ± 61 61 ± 19	778 ± 65 65 ± 38	806 ± 60 60 ± 16	876 ± 104 104 ± 81	817 ± 58 58 ± 5
GSR mean [µS] SCL mean [µS]	0.07 ± 0.05 0.06 ± 0.04	0.17 ± 0.09 0.10 ± 0.08	0.12 ± 0.10 0.10 ± 0.09	0.13 ± 0.10 0.11 ± 0.09	0.14 ± 0.11 0.12 ± 0.11

**Table 2:** Mean number of peaks of the SCR component during the five considered time windows averaged on all the subjects.

Time Window	W1 (R1 End)	W2 (SF Start)	W3 (R2 Start)	W4 (R2 End)	W5 (BH Start)
No of SCR peaks	1.3	5.3	2.7	1.3	3.8

## Data Availability

The data that support the findings of this study are available upon request from the authors.
